# Pennsylvania’s Annual Meeting

**Published:** 1898-04

**Authors:** 


### PENNSYLVANIA'S ANNUAL MEETING.

PENNSYLVANIA held in March one of the largest and most successful State veterinary meetings ever held in the history of the profession. Over one hundred and thirty veterinarians were present at the several sessions of the meeting, and the greatest interest was evidenced on all sides pertaining to the work of our profession. Excellent reports, papers, and lectures were presented, and, with a sumptuous banquet spread for the guests by Dr. Leonard Pearson, it was, all in all, one of the great pleasures of these times to have been there.

